# To be or not to B27 positive: implications for the phenotypes of axial spondyloarthritis outcomes. Data from a large multiracial cohort from the Brazilian Registry of Spondyloarthritis

**DOI:** 10.1186/s42358-024-00372-0

**Published:** 2024-04-26

**Authors:** Gustavo Gomes Resende, Carla Goncalves Schahin Saad, Claudia Diniz Lopes Marques, Sandra Lúcia Euzébio Ribeiro, Maria Bernadete Renoldi de Oliveira Gavi, Michel Alexandre Yazbek, Adriana de Oliveira Marinho, Rita de Cássia Menin, Manuella Lima Gomes Ochtrop, Andressa Miozzo Soares, Nara Gualberto Cavalcanti, Jamille Nascimento Carneiro, Glaucio Ricardo Werner de Castro, José Mauro Carneiro Fernandes, Elziane da Cruz Ribeiro E Souza, Corina Quental de Menezes Alvarenga, Rejane Maria Rodrigues de Abreu Vieira, Natalia Pereira Machado, Antônio Carlos Ximenes, Morgana Ohira Gazzeta, Cleandro Pires de Albuquerque, Thelma Larocca Skare, Mauro Waldemar Keiserman, Charles Lubianca Kohem, Gabriel Sarkis Benacon, Vítor Florêncio Santos Rocha, Ricardo da Cruz Lage, Olivio Brito Malheiro, Rywka Tenenbaum Medeiros Golebiovski, Thauana Luiza Oliveira, Ruben Horst Duque, Ana Carolina Londe, Marcelo de Medeiros Pinheiro, Percival Degrava Sampaio-Barros

**Affiliations:** 1 Hospital das Clínicas da UFMG, Belo Horizonte, Brazil; 2 Faculdade de Medicina da USP, São Paulo, Brazil; 3 Hospital das Clínicas da UFPE, Recife, Brazil; 4 Faculdade de Medicina da UFAM, Manaus, Brazil; 5 Universidade Federal do Espírito Santo, Vitória, Brazil; 6 Universidade Estadual de Campinas, Campinas, Brazil; 7 Fundação Hospital do Acre, Rio Branco, Brazil; 8 Faculdade de Medicina de São José do Rio Preto, São José do Rio Preto, Brazil; 9 Universidade Estadual do Rio de Janeiro, Rio de Janeiro, Brazil; 10 Hospital Universitário da UFSC, Florianópolis, Brazil; 11 Hospital de Base do Distrito Federal, Brasília, Brazil; 12 Hospital Governador Celso Ramos, Florianópolis, Brazil; 13 Universidade Federal do Maranhão, São Luís, Brazil; 14 Hospital de Clínicas da UFU, Uberlândia, Brazil; 15 Santa Casa de Belo Horizonte, Belo Horizonte, Brazil; 16 Universidade Estadual do Ceará, Fortaleza, Brazil; 17 Universidade Federal do Paraná, Curitiba, Brazil; 18 Hospital Geral de Goiânia, Goiânia, Brazil; 19 Santa Casa do Rio de Janeiro, Rio de Janeiro, Brazil; 20 Hospital Universitário de Brasília, Brasília, Brazil; 21 Hospital Universitário Evangélico Mackenzie, Curitiba, Brazil; 22 Faculdade de Medicina da PUCRS, Porto Alegre, Brazil; 23 Faculdade de Medicina da UFRGS, Porto Alegre, Brazil; 24 Escola Paulista de Medicina – UNIFESP, São Paulo, Brazil; 25 Serviço de Reumatologia do Hospital das Clínicas da Universidade Federal de Minas Gerais, Alameda Álvaro Celso, 175/2nd floor, Santa Efigênia, Belo Horizonte, MG Brazil

**Keywords:** Axial spondyloarthritis, HLA-B27, Genetics, Register-study, ASDAS-CRP, Ankylosing spondylitis

## Abstract

**Background:**

There is a remarkable variability in the frequency of HLA-B27 positivity in patients with spondyloarthritis (SpA), which may be associated with different clinical presentations worldwide. However, there is a lack of data considering ethnicity and sex on the evaluation of the main clinical and prognostic outcomes in mixed-race populations. The aim of this study was to evaluate the frequency of HLA-B27 and its correlation with disease parameters in a large population of patients from the Brazilian Registry of Spondyloarthritis (RBE).

**Methods:**

The RBE is a multicenter, observational, prospective cohort that enrolled patients with SpA from 46 centers representing all five geographic regions of Brazil. The inclusion criteria were as follow: (1) diagnosis of axSpA by an expert rheumatologist; (2) age ≥18 years; (3) classification according to ASAS axial. The following data were collected via a standardized protocol: demographic data, disease parameters and treatment historical.

**Results:**

A total of 1096 patients were included, with 73.4% HLA-B27 positivity and a mean age of 44.4 (±13.2) years. Positive HLA-B27 was significantly associated with male sex, earlier age at disease onset and diagnosis, uveitis, and family history of SpA. Conversely, negative HLA-B27 was associated with psoriasis, higher peripheral involvement and disease activity, worse quality of life and mobility.

**Conclusions:**

Our data showed that HLA-B27 positivity was associated with a classic axSpA pattern quite similar to that of Caucasian axSpA patients around the world. Furthermore, its absence was associated with peripheral manifestations and worse outcomes, suggesting a relevant phenotypic difference in a highly miscegenated population.

## Background

Since its association with ankylosing spondylitis (AS) was discovered 50 years ago [1, 2], HLA-B27 has been considered an important genetic marker and also plays a significant role in the 2009 classification criteria for axial spondyloarthritis (axSpA) [3]. Early epidemiological studies showed that HLA-B27 was more prevalent in white populations in Northern Europe, which also had a greater prevalence of axSpA [4]. In the same context, the heterogeneity in the prevalence and clinical presentation of SpA observed in different regions of the world seems to be associated with the prevalence of HLA-B27 in these populations [5].

Clinically, HLA-B27 is associated with axial involvement and acute anterior uveitis in patients with a younger age at onset and a positive family history of axSpA [6, 7]. The association with worse radiographic progression in axSpA patients is another important characteristic [8]. Recently, HLA-B27 has also been shown to be an important phenotypic marker in peripheral spondyloarthritis (SpA); in the ASAS PerSpA study, the presence of HLA-B27 was associated with male sex, earlier age at onset, and the presence of axial involvement, tarsitis, and uveitis even in patients with peripheral SpA [9]. Its potential association with a better response to treatment in axSpA patients was also demonstrated [10].

Progression to axSpA and mortality can also be associated with HLA-B27. A Dutch study showed that the progression to axSpA at the 5-year-follow-up in first degree relatives of patients with axSpA was greater only in the HLA-B27-positive group in a pre-SpA cohort [11]. The lifetime recurrence rate of axSpA appears to be greater in first-degree relatives of HLA-B27 patients, and interestingly, affected mothers can transfer the disease more frequently to their offspring than can their affected fathers [12]. A recent study evaluating the 35-year-follow-up of a British axSpA cohort revealed increased mortality associated with HLA-B27, especially in women, in patients with radiographic axSpA, but not in patients with non-radiographic axSpA [13]. Recent findings emphasize that analysis of axSpA patients may also need to consider the distinct profile of the disease in women [14–16].

Latin America, especially Brazil, is traditionally a region of remarkable racial miscegenation. In addition to the original indigenous population, the White from Europe, Blacks brought from Africa, and later Asian populations comprise a large multiracial nation in Brazil. This racial diversity is associated with the heterogeneity of the clinical presentation of SpA in the Brazilian population [17], a finding quite similar to that demonstrated in North American individuals [18, 19]. Recently, a national Brazilian study analyzing more than 5 million healthy bone marrow donors showed a prevalence of 4.35% of HLA-B27 positivity in the Brazilian population, ranging from 4.85% in Whites to 2.92% in nonmiscegenate Blacks [20].

Therefore, the present study evaluated the frequency of HLA-B27 and its clinical-epidemiological associations in a large population of patients with axSpA from the Brazilian Registry of Spondyloarthritis (*Registro Brasileiro de Espondiloartrites—*RBE).

## Methods

### Design

The RBE is a multicenter, observational, prospective cohort that enrolled patients with SpA from 46 centers representing all five main geographic regions of Brazil from 2018 to 2023. To participate in this study, patients needed to meet the following inclusion criteria (1): had a diagnosis of axSpA by an expert rheumatologist (2); were aged older than 18 years; and (3) were classified according to the ASAS axial criteria [3].

Data, which included demographic data (age, sex, skin color, work situation), positivity of HLA-B27 and disease parameters (axial and/or peripheral involvement, extramusculoskeletal manifestations—EMM, comorbidities and treatment), were collected in a standardized protocol on the REDCap platform.

### Disease outcomes

For patients with axSpA, the Bath Ankylosing Spondylitis Disease Activity Index (BASDAI) [21] and Ankylosing Spondylitis Disease Activity Score (ASDAS) [22] using erythrocyte sedimentation rate (ESR) and C-reactive protein (CRP) were used to measure disease activity. Other indices, such as Bath Ankylosing Spondylitis Functional Index (BASFI) [23], Bath Ankylosing Spondylitis Metrology Index (BASMI) [24], Ankylosing Spondylitis Quality of Life (ASQoL) [25], and Maastricht Ankylosing Spondylitis Enthesitis Score (MASES) [26] were also measured.

### Ethics

The study was performed in accordance with the principles of the Declaration of Helsinki and approved by the Institutional Ethical Committee (Comissão de Ética para Análise de Projetos de Pesquisa—CAPPesq) of Hospital das Clinicas HCFMUSP, Faculdade de Medicina, Universidade de Sao Paulo, Brazil (ID CAAE: 49299415.7.1001.0068). Written informed consent was obtained from all participants.

### Statistical analysis

To characterize the participants’ profile, absolute and relative frequencies, means and standard deviations (SDs) were calculated. Comparisons of means between two groups were performed using Student’s t test for independent samples. To verify the normality of the data, Kolmogorov-Smirnov test was applied. In the case of normality violation, Mann-Whitney non-parametric test was used. The chi-square test (or Fischer’s exact test for small samples) were used to evaluate associations among categorical variables.

A logistic regression model in which HLA-B27 positivity was used as the dependent variable and appropriate adjustments were performed considering all independent variables that had a statistical significance of up to 10% in the univariate analysis. The P value was considered significant if it was less than 5%. Multiple linear regression models were also constructed for the main outcomes in patients with axSpA (ASDAS-CRP, BASDAI, BASFI, BASMI, and ASQoL). Statistical analyzes were performed using the IBM SPSS Statistics for Windows (Version 27.0. Armonk, NY: IBM Corp and GraphPad Software, Boston, Massachusetts USA, version 10) and RStudio 2023.06.1+524 “Mountain Hydrangea” for Windows.

## Results

Among the 1392 patients with SpA registered in the RBE, 1096 had axSpA, with 73.4% of HLA-B27 positivity. It was notably associated with male sex (*p* = 0.004), younger age at onset of symptoms (*p* < 0.0001) and at diagnosis (*p* < 0.0001), family history of SpA (*p* = 0.03), uveitis manifestation (*p* = 0.0001), and lower prevalence of psoriasis (*p* < 0.0001). Furthermore, the axSpA patients who were HLA-B27 negative had greater disease activity, as measured by the BASDAI (*p* = 0.02) and ASDAS (*p* = 0.004); worse quality of life, as assessed by the ASQoL (*p* = 0.03), more impaired mobility, as assessed by the BASMI (*p* = 0.045) and more frequent peripheral involvement, as assessed by the painful joint count (*p* = 0.007). See Table 1.

**Table 1:** Demographic and clinical features of axial SpA patients included in the RBE

Characteristic	Total population (n = 1096)	HLA-B27 positive (n = 805)	HLA-B27 negative (n = 291)	p Value
Male gender (n, %)	803 (73.3)	609 (75.7)	194 (66.7)	**0.004**
Disease duration (years, SD)	18.3 (12.0)	18.6 (12.5)	17.4 (10.8)	0.485
Mean age at symptoms onset (years, SD)	28.4 (12.0)	27.0 (11.6)	32.2 (12.2)	**< 0.001**
Mean age at diagnosis (years, SD)	34.2 (12.4)	32.7 (11.7)	38.1 (12.5)	**< 0.001**
Mean delay in diagnosis (years, SD)	9.1 (9.3)	9.1 (9.7)	9.3 (8.4)	0.284
Race (self-reported skin color)				0.121
White (n, %)	537 (50.9)	412 (53.0)	125 (44.8)	
Black (n, %)	76 (7.2)	52 (6.7)	24 (8.6)	
Brown (*Pardos*) (n, %)	387 (36.6)	271 (34.9)	116 (41.6)	
Yellow (Asian Brazilians) (n, %)	38 (3.6)	30 (3.9)	8 (2.9)	
Indigenous (n, %)	18 (1.7)	12 (1.5)	6 (2.2)	
BMI (mean, SD)	26.5 (4.8)	26.4 (4.6)	26.8 (5.2)	0.493
Family history of SpA (n, %)	109 (22.1)	86 (24.7)	23 (15.8)	**0.032**
EMMs				**< 0.001**
Any (n, %)	231 (34.8)	170 (35.4)	61 (33.3)	
Psoriasis (n, %)	39 (5.9)	15 (3.1)	24 (13.1)	
IBD (n, %)	30 (4.5)	14 (2.9)	16 (8.7)	
Uveitis (n, %)	176 (26.5)	147 (30.6)	29 (15.8)	
Painful enthesis count (mean, SD)	5.0 (4.7)	5.0 (4.8)	5.1 (4.5)	0.554
Painful joint count (mean, SD)	6.9 (10.5)	5.7 (8.7)	9.8 (13.5)	**0.007**
Swollen joint count (mean, SD)	3.6 (4.2)	3.3 (4.0)	4.3 (4.5)	0.24
BASDAI (mean, SD)	4.1 (2.5)	4.0 (2.5)	4.3 (2.4)	**0.019**
ASDAS-CRP (mean, SD)	3.4 (1.7)	3.3 (1.7)	3.7 (1.8)	**0.004**
ASQoL (mean, SD)	7.5 (5.6)	7.3 (5.7)	8.1 (5.4)	**0.034**
BASMI (mean, SD)	3.9 (2.1)	3.8 (2.1)	4.2 (2.1)	**0.045**
BASFI (mean, SD)	4.6 (2.9)	4.5 (2.9)	4.8 (2.8)	0.059

The results are expressed as the mean (SD) and n (%); *BMI* body mass index, *EMM* extra-articular manifestation, *IBD* inflammatory bowel disease, *BASDAI* bath ankylosing spondylitis disease activity index, *ASDAS–CRP* axial spondyloarthritis disease activity score-C reactive protein, *ASQoL* ankylosing spondylitis quality of life, *BASMI* bath ankylosing spondylitis metrology index, *BASFI* bath ankylosing spondylitis functional index, *SD* standard deviation

p-values in bold represent statistically significant findings

According to the multivariate analysis, the final logistic regression model (with HLA-B27 as the dependent variable) showed a significant association between positivity for HLA-B27 and male sex (OR = 1.49; 95%CI = 1.01–2.35, *p* = 0.02), earlier age at disease onset (*p* = 0.01), the presence of uveitis (OR = 1.62; 95%CI = 1.05–2.79, *p* = 0.02) and the absence of psoriasis (OR = 0.25; 95%CI = 0.11–0.58, *p* = 0.001). Furthermore, in patients with axSpA, the absence of HLA-B27 was associated with increased disease activity, as measured by the ASDAS-CRP (*p* = 0.026). OR should be used solely to understand the effect size of the relationship between categorical variables. In this passage, there is no OR because ASDAS was not treated as categorical but rather as numerical, which went unnoticed during the review process.

To better understand the aforementioned association observed between HLA-B27 and lower ASDAS-CRP in axSpA patients, we explored several linear regression models with axSpA outcome measures (ASDAS-CRP, BASDAI, BASFI, BASMI, and ASQoL) as the dependent variables. The optimal fit model demonstrated an association between higher ASDAS-CRP scores and absence of HLA-B27 (*p* = 0.014), along with male sex (*p* = 0.006), older age (*p* = 0.002), shorter duration of symptoms (*p* = < 0.001), increased painful joint count (*p* = 0.007), and more frequent use of current NSAIDs (*p* = 0.001). Among the other axSpA outcome measures, only BASDAI showed an association with the absence of HLA-B27 (*p* = 0.046). Table 2 shows the final multivariate linear regression models for all the main outcomes in patients with axSpA.

**Table 2:** Multivariate linear regression models for the main outcomes in axial SpA patients

Dependent variable	Independent variables associated, according to the best-fitting model										
**ASDAS-CRP**	Male sex ***P*** **= 0.006** **B = 0.13**	Older age ***P*** **= 0.002** **B = 0.16**	Shorter duration of symptoms ***P*** **< 0.0001** **B = 0.20**	Higher NSAID prescriptions ***P*** **= 0.001** **B = 0.16**	HLA-B27 Absent ***P*** **= 0.014** **B = 0.12**	Increased PJC ***P*** **= 0.007** **B = 0.13**	–	–	–	–	–
**BASDAI**	–	–	–	–	HLA-B27 Absent ***P*** **= 0.046** **B = 0.10**	Increased PJC ***P*** **< 0.0001** **B = 0.07**	Longer diagnostic delay ***P*** **= 0.0004** **B = 0.18**	Increased enthesis count ***P*** **< 0.0001** **B = 0.29**	–	–	–
**BASMI**	–	–	Longer duration of symptoms ***P*** **< 0.0001** **B = 0.35**	Higher NSAID prescriptions ***P*** **= 0.01** **B = 0.12**	–	–	Longer diagnostic delay ***P*** **< 0.0001** **B = 0.26**	–	BASDAI ***P*** **< 0.0001** **B = 0.24**	–	–
**BASFI**	–	Older age ***P*** **= 0.03** **B = 0.11**	Longer duration of symptoms ***P*** **= 0.0001** **B = 0.19**	–	–	Increased PJC ***P*** **= 0.001** **B = 0.14**	Longer diagnostic delay ***P*** **= 0.02** **B = 0.11**	–	–	BASMI ***P*** **< 0.0001** **B = 0.54**	–
**ASQoL**	–	–	–	–	–	–	–	–	BASDAI ***P*** **< 0.0001** **B = 0.30**	–	BASFI ***P*** **< 0.0001** **B = 0.29**

*ASDAS-CRP* axial spondyloarthritis disease activity score (C-reactive protein), *BASDAI* bath ankylosing spondylitis disease activity index, *ASQoL* ankylosing spondylitis quality of life, *BASMI* bath ankylosing spondylitis metrology index, *BASMI* bath ankylosing spondylitis metrology index, *NSAID* nonsteroidal anti-inflammatory drug, *PJC* painful joints count, *P* p value, *B* linear regression standardized coefficients

Bold formatting is intended solely to highlight the p-values and standardized B coefficients that were significant in each model (row)

## Discussion

At this time when new biomarkers were first discovered in axSpA patients half a century after the discovery of the association between HLA-B27 and AS [27], it is important to understand the entire spectrum of disease associated with both the presence and absence of HLA-B27 in different populations worldwide. With the expansion of the concept of axSpA, HLA-B27 research has become even more valuable for assisting in both the diagnosis and prognosis of this disease [3, 28]. This study in a significantly heterogeneous sample of the Brazilian population showed that while the presence of HLA-B27 was associated with the classic phenotype observed in patients from more homogeneous populations in Europe and North America, the absence of HLA-B27 seems to be associated with the peripheral manifestations of axSpA and worse outcome measures, a striking characteristic of miscegenated Latin-American populations.

Positivity for HLA-B27 was observed in 73.4% of the patients with axSpA. This finding is quite similar to that observed in a recent compilation of Brazilian studies with a grouped frequency of approximately 75% but with some variability between different geographic regions related to colonization, migration, culture and origins [20]. This relevant heterogeneity was also noted by another study comparing SpA patients from the Latin American Registry of SpA (RESPONDIA) with those from two European registries (the Spanish REGISPONSER and the Belgian GIANT) with a significantly lower prevalence of HLA-B27 in Latin-Americans (71% vs. 83%, respectively) [29]. A subsequent study analyzing 4067 patients from REGISPONSER and RESPONDIA showed that HLA-B27 negative patients had more peripheral involvement as the initial manifestation of SpA [30]. A recent publication evaluating 5557 patients with axSpA, members of an international patient network, revealed that Latin America was the continent with the lowest proportion of positive HLA-B27 (65%), ranging on other continents from 71% in Europe to 78% in Asia [31]. Corroborating this fact, an Argentinian study found a lower proportion of HLA-B27 (43%) in patients with axSpA [32].

Although HLA-B27 has been significantly associated with White ancestry, especially of Caucasian origin, the findings regarding HLA-B27 in the Brazilian population of African descent with axSpA are especially interesting. In the present study, 463 (42.2% of the total axSpA patients) are of African descent, most of whom are brown, with only 7.2% being nonmiscegenated Blacks. Even so, HLA-B27 positivity was 70% in brown patients and 68.4% in Black patients. A previous study evaluating the prevalence of HLA-B27 in three ethnic groups in the United States showed that the frequency of HLA-B27 positivity was significantly lower in Blacks (62.5%) than in Whites (85.3%) [33]. On the other hand, as the prevalence of HLA-B27 is very low (<1%) in Black populations in sub-Saharan Africa, rare descriptions of the frequency of HLA-B27 in these populations have been reported, predominantly with a very small number of patients [34]. More recently, a systematic review showed an unmet need regarding the paucity of population-data available to estimate it for the global SpA population [35].

From 1500 to 1889 (the year slavery was abolished in Brazil), more than nine million people were brought from Africa to the Americas. It is important to highlight that there is a correspondence between the geographic origin of different regions of Africa and certain destinations of the diaspora in the Americas, characterizing the differences in ancestry and miscegenation with relevant genetic diversity from the New World. Central-western regions of Africa, such as Nigeria, Senegal, Ghana, Gambia and Kenya, had a greater proportion of people taken to the Caribbean and North America, while Bantu people from southern and eastern Africa (Sub-Saharan) were more often taken to Brazil [36]. These ancestry details could explain the differences observed even in individuals of Black origin spread across the Americas. Thus, our study contributes to a better understanding of how HLA-B27 could impact axSpA outcomes in a miscegenated population.

Previous studies in Latin America have demonstrated that the clinical presentation of SpA may differ depending on the patient´s sex [37, 38]. Subsequent studies, after the publication of the SpA concept, have demonstrated that clinical investigation axSpA in women deserves to have a specific focus [14–16]. The present study confirmed that HLA-B27 was significantly less frequent in women. Additional and more comprehensive analyzes are needed the evaluation of female patients with axSpA.

A significantly earlier age at disease onset (5.2 years difference) was another important finding in this study, according to both univariate and multivariate analyses. These data confirm the findings of the PerSpA study, which revealed a significant 6-year difference in disease onset in HLA-B27 positive patients [39]. A post-hoc analysis of 2910 patients from the ASAS PerSpA study revealed that the HLA-B27 negative group of patients had a more delayed diagnosis, as well as a greater prevalence of peripheral arthritis and enthesitis, in addition to extra-articular manifestations [40].

The significant associations of negative HLA-B27 with worse outcome measures (BASDAI, ASDAS, ASQoL, and BASMI in univariate analysis; and only BASDAI and ASDAS in multivariate analysis) in patients with axSpA also deserve consideration. A recent review on the characterization of negative HLA-B27 revealed similar results, but disease activity was measured by ESR and CRP [41] and not by the outcome measures recommended by the ASAS [42]. It is important to highlight the fact that although both the BASDAI and ASDAS were associated with a greater PJC, only BASDAI was associated with enthesitis (Table 2), probably because it has a specific question about entheses (question 4: “How would you describe the overall level of discomfort you have had in the past week from any areas tender to touch or pressure?”) in its questionnaire.

Addressing the racial context, the North-South gradient observed in the healthy Brazilian population is also notable, where HLA-B27 becomes increasingly more frequent as we migrate from the North (with a predominantly miscegenated population) to the South (with a majority population of White European ancestry) [20]. Although the present study was not designed for this specific evaluation, we can infer that the association between not having HLA-B27 and higher ASDAS-CRP may be associated with greater peripheral involvement in patients with axSpA, regardless of functionality, mobility, or quality of life impairment. Thus, our data highlighted another unmet need in managing patients with combined axial and peripheral involvement.

Regarding EMMs, this study showed that while uveitis is significantly associated with the presence of HLA-B27, psoriasis is associated with its absence. The association of HLA-B27 with anterior uveitis is well known [43], and it has become increasingly common to manifest at the onset of axSpA [44]. The presence of psoriasis tends to be more frequent in HLA-B27-negative patients, even in early SpA cohorts [45], and it can be inferred that the presence of HLA-B27 reduces the chance of concomitant diagnosis of psoriasis [45, 46].

The main strength of this study is the possibility of using a representative population from the different geographic regions of the country, allowing us to evaluate the ethnic diversity of the Brazilian population, with half of the patients being non-white, similar to what is observed in the last geographic census of the Brazilian population [47]. The main weakness of our study, as frequently occurs in the evaluation of highly miscegenated populations, is the characterization of ethnicity; we chose to consider the skin color that patients attribute to themselves.

## Conclusions

In a scenario where there are still unmet needs regarding the diagnosis and classification of axSpA [48], the study of different populations will help to better understand the distinct phenotypes of axSpA. As HLA-B27 can be associated with somewhat different phenotypes, according to its presence or absence, we can assume that HLA-B27 may be one of the factors associated with the heterogeneous presentation of axSpA in Brazil, a clearly miscegenated nation.

## Data Availability

The authors confirm that the de-identified data supporting the findings of this study can be made available to others on approval of a written reasonable request to the corresponding author.

## List of abbreviations

AS: Ankylosing spondylitis
ASAS: Assessment of spondyloarthritis international society
ASDAS: Axial spondyloarthritis disease activity score
ASQoL: Ankylosing spondylitis quality of life
axSpA: Axial spondyloarthritis
BASDAI: Bath ankylosing spondylitis disease activity index
BASFI: Bath ankylosing spondylitis functional index
BASMI: Bath ankylosing spondylitis metrology index
BMI: Body mass index
bDMARD: Biological disease modifying anti-rheumatic drug
CRP: C-reactive protein
DMARDs: Disease modifying anti-rheumatic drug
EMM: Extramusculoskeletal manifestations
ESR: Erythrocyte sedimentation rate
MASES: Maastricht ankylosing spondylitis enthesitis score
IBD: Inflammatory bowel disease
NSAID: Nonsteroidal anti-inflammatory drug
RBE: Brazilian Registry of Spondyloarthritis
SD: Standard deviations
SpA: Spondyloarthritis

## References

[1] Brewerton DA, Hart FD, Nicholls A, Caffrey M, James DC, Sturrock RD. Ankylosing spondylitis and HL-A 27. Lancet. 1973;1(7809):904–07. 4123836 10.1016/s0140-6736(73)91360-3

[2] Schlosstein L, Terasaki PI, Bluestone R, Pearson CM. High association of an HL-A antigen, W27, with ankylosing spondylitis. N Engl J Med. 1973;288(14):704–06. 4688372 10.1056/NEJM197304052881403

[3] Rudwaleit M, van der Heijde D, Landewe R, Listing J, Akkoc N, Brandt J, et al. The development of assessment of spondyloarthritis international society classification criteria for axial spondyloarthritis (part II): validation and final selection. Ann Rheum Dis. 2009;68:777–83. 19297344 10.1136/ard.2009.108233

[4] Stolwijk C, Boonen A, van Tubergen A, Reveille JD. Epidemiology of spondyloarthritis. Rheum Dis Clin North Am. 2012;38(3):441–76. 23083748 10.1016/j.rdc.2012.09.003 PMC4470267

[5] Fahed H, Mauro D, Ciccia F, Ziade NR. What does human leukocyte antigen B27 have to do with spondyloarthritis? Rheum Dis Clin North Am. 2020;46(2):225–39. 32340698 10.1016/j.rdc.2020.01.002

[6] Bittar M, Yong WC, Magrey M, Khan MA. Worldwide differences in clinical phenotype of axial spondyloarthritis. Curr Rheumatol Rep. 2021;23(10):76. 34586533 10.1007/s11926-021-01043-5

[7] Ziade N. Human leucocyte antigen-B27 testing in clinical practice: a global perspective. Curr Opin Rheumatol. 2023;35(4):235–42. 37115941 10.1097/BOR.0000000000000946

[8] Coates LC, Baraliakos X, Blanco FJ, Blanco-Morales EA, Braun J, Chandran V, et al. The phenotype of axial spondyloarthritis: is it dependent on HLA-B27 status? Arthritis Care Res (Hoboken). 2021;73:856–60. 32100954 10.1002/acr.24174

[9] Arevalo Salaet M, López-Medina C, Moreno M, Navarro-Compan V, Calvet Fontova J, Llop M, et al. Association between HLA-B27 and peripheral spondyloarthritis phenotype: results from the ASAS perSpA study. RMD Open. 2022;8:e002696. 36597968 10.1136/rmdopen-2022-002696 PMC9743377

[10] Fröhlich F, Micheroli R, Hebeisen M, Kissling S, Bürki K, Exer P, et al. HLA-B27 as a predictor of effectiveness of treatment with TNF inhibitors in axial spondyloarthritis: data from the Swiss clinical quality management registry. Clin Rheumatol. 2023;42:1267–74. 36574181 10.1007/s10067-022-06490-8 PMC10102047

[11] de Jong HMY, de Winter JJH, van der Horst-bruinsma IE, van Schaardenburg DJ, van Gaalen FA, van Tubergen AM, et al. Progression from subclinical inflammation to overt spondyloarthritis in first-degree relatives of patients in association with HLA-B27: the pre-spondyloarthritis cohort. Arthritis Care Res (Hoboken). 2022;74:2076–84. 34219406 10.1002/acr.24743 PMC10087210

[12] van der Linden SM, Khan MA, Li Z, Baumberger H, Zandwijk HV, Khan MK, et al. Recurrence of axial spondyloarthritis among first-degree relatives in a prospective 35-year-follow-up family study. RMD Open. 2022;8:e002208. 35868737 10.1136/rmdopen-2022-002208 PMC9315900

[13] Li Z, Khan MK, van der Linden SM, Winkens B, Villiger PM, Baumberger H, et al. HLA-B27, axial spondyloarthritis and survival. Ann Rheum Dis. 2023;82:1558–67. 37679034 10.1136/ard-2023-224434

[14] Chimenti MS, Alten R, D’Agostino MA, Gremese E, Kiltz U, Lubrano E, et al. Sex-associated and gender-associated differences in the diagnosis and management of axial spondyloarthritis: addressing the unmet needs of female patients. RMD Open. 2021;7:e001681. 34876490 10.1136/rmdopen-2021-001681 PMC8655606

[15] Li Z, van der Linden SM, Khan MA, Baumberger H, Zandwijk HV, Khan MK, et al. Heterogeneity of axial spondyloarthritis: genetics, sex and structural damage matter. RMD Open. 2022;8:e002302. 35523521 10.1136/rmdopen-2022-002302 PMC9083385

[16] Ensslin C, Micheroli R, Kissling S, Götschi A, Bürki K, Bräm R, et al. Impact of sex on spinal radiographic progression in axial spondyloarthritis: a longitudinal Swiss cohort analysis over a period of 10 years. RMD Open. 2023;9:e003340. 37507208 10.1136/rmdopen-2023-003340 PMC10387740

[17] Saad CG, Gonçalves CR, Sampaio-Barros PD. Seronegative arthritis in Latin America: a current review. Curr Rheumatol Rep. 2014;16(9):438. 25023724 10.1007/s11926-014-0438-3

[18] Khan MA, Braun WE, Kushner I, Grecek DE, Muir WA, Steinberg AG. HLA B27 in ankylosing spondylitis: differences in frequency and relative risk in American blacks and caucasians. J Rheumatol Suppl. 1977;3:39–43. 266595

[19] Reveille JD, Weisman MH. The epidemiology of back pain, axial spondyloarthritis and HLA-B27 in the United States. Am J Med Sci. 2013;345(6):431–36. 23841117 10.1097/maj.0b013e318294457f PMC4122314

[20] Resende GG, Saad CGS, de Oliveira DCM, de Sousa Bueno Filho JS, Sampaio-Barros PD, de Medeiros Pinheiro M. HLA-B27 positivity in a large miscegenated population of 5,389,143 healthy blood marrow donors in Brazil. Adv Rheumatol. 2023;63(1):16. 37081582 10.1186/s42358-023-00302-6

[21] Garrett S, Jenkinson T, Kennedy LG, Whitelock H, Gaisford P, Calin A. A new approach to defining disease status in ankylosing spondylitis: the bath ankylosing spondylitis disease activity index. J Rheumatol. 1994;21(12):2286–91. 7699630

[22] Lukas C, Landewe R, Sieper J, Dougados M, Davis J, Braun J, et al. Development of an ASAS-endorsed disease activity score (ASDAS) in patients with ankylosing spondylitis. Ann Rheum Dis. 2009;68:18–24. 18625618 10.1136/ard.2008.094870

[23] Calin A, Garrett S, Whitelock H, Kennedy LG, O’Hea J, Mallorie P, et al. A new approach to defining functional ability in ankylosing spondylitis: the development of the bath ankylosing spondylitis functional index. J Rheumatol. 1994;21:2281–85. 7699629

[24] Jones SD, Porter J, Garrett SL, Kennedy LG, Whitelock H, Calin A. A new scoring system for the bath ankylosing spondylitis metrology index (BASMI). J Rheumatol. 1995;22(8):1609. 7473496

[25] Doward LC, Spoorenberg A, Cook SA, Whalley D, Helliwell PS, Kay LJ, et al. Development of the ASQoL: a quality of life instrument specific to ankylosing spondylitis. Ann Rheum Dis. 2003;62:20–26. 12480664 10.1136/ard.62.1.20 PMC1754293

[26] Heuft-Dorenbosch L, Spoorenberg A, van Tubergen A, Landewé R, van Ver Tempel H, Mielants H, et al. Assessment of enthesitis in ankylosing spondylitis. Ann Rheum Dis. 2003;62:127–32. 12525381 10.1136/ard.62.2.127 PMC1754445

[27] Khan MA. HLA-B*27 and ankylosing spondylitis: 50 years of insights and discoveries. Curr Rheumatol Rep. 2023;25(12):327–40. 37950822 10.1007/s11926-023-01118-5

[28] van der Heijde D, Molto A, Ramiro S, Braun J, Dougados M, van Gaalen FA, et al. Goodbye to the term ‘ankylosing spondylitis’, hello ‘axial spondyloarthritis’: time to embrace the ASAS-defined nomenclature. Ann Rheum Dis. 2023/2024;83:547–49. 10.1136/ard-2023-225185 38071514

[29] Benegas M, Muñoz-Gomariz E, Font P, Burgos-Vargas R, Chaves J, Palleiro D, et al. Comparison of the clinical expression of patients with ankylosing spondylitis from Europe and Latin America. J Rheumatol. 2012;39:2315–20. 23149388 10.3899/jrheum.110687

[30] Puche-Larrubia M, Ladehesa-Pineda L, Vázquez-Mellado J, Escudero-Contreras A, Gratacós J, Juanola X, et al. Identification of the first signs or symptoms in different spondyloarthritis subtypes and their association with HLA-B27: data from REGISPONSER and RESPONDIA registries. RMD Open. 2023;9:e003235. 37734875 10.1136/rmdopen-2023-003235 PMC10514611

[31] Poddubnyy D, Sommerfleck F, Navarro-Compán V, Bundy C, Makri S, Akerkar S, et al. Regional differences in clinical phenotype of axial spondyloarthritis: results from the international map of axial spondyloarthritis (IMAS). Rheumatology (Oxford). 2023;Dec 21:kead665. Epub ahead of print. 10.1093/rheumatology/kead665 PMC11371368 38128022

[32] García-Salinas R, Ruta S, Chichande JT, Magri S. The role of HLA-B27 in Argentinian axial spondyloarthritis patients. J Clin Rheumatol. 2022;28(2):e619–e622. 34145202 10.1097/RHU.0000000000001763

[33] Jamalyaria F, Ward MM, Assassi S, Learch TJ, Lee M, Gensler LS, et al. Ethnicity and disease severity in ankylosing spondylitis a cross-sectional analysis of three ethnic groups. Clin Rheumatol. 2017;36:2359–64. 28780639 10.1007/s10067-017-3767-6 PMC5693696

[34] Tikly M, Njobvu P, McGill P. Spondyloarthritis in sub-Saharan Africa. Curr Rheumatol Rep. 2014;16(6):421. 24744085 10.1007/s11926-014-0421-z

[35] Bohn R, Cooney M, Deodhar A, Curtis JR, Golembesky A. Incidence and prevalence of axial spondyloarthritis: methodologic challenges and gaps in the literature. Clin Exp Rheumatol. 2018;36(2):263–74. 29148402

[36] Gouveia MH, Borda V, Leal TP, Moreira RG, Bergen AW, Kehdy FSG, et al. Origins, admixture dynamics, and homogenization of the African gene pool in the Americas. Mol Biol Evol. 2020;37:1647–56. 32128591 10.1093/molbev/msaa033 PMC7253211

[37] de Carvalho HM, Bortoluzzo AB, Goncalves CR, da Silva JA, Ximenes AC, Bertolo MB, et al. Gender characterization in a large series of Brazilian patients with spondyloarthritis. Clin Rheumatol. 2012;31:687–95. 22203094 10.1007/s10067-011-1890-3

[38] Landi M, Maldonado-Ficco H, Perez-Alamino R, Maldonado-Cocco JA, Citera G, Arturi P, et al. Gender differences among patients with primary ankylosing spondylitis and spondylitis associated with psoriasis and inflammatory bowel disease in an iberoamerican spondyloarthritis cohort. Medicine (Baltimore). 2016;95:e5652. 28002334 10.1097/MD.0000000000005652 PMC5181818

[39] Boel A, van Lunteren M, López-Medina C, Sieper J, van der Heijde D, van Gaalen FA. Geographical prevalence of family history in patients with axial spondyloarthritis and its association with HLA-B27 in the ASAS-PerSpA study. RMD Open. 2022;8(1):e002174. 35301267 10.1136/rmdopen-2021-002174 PMC8932257

[40] Chaudhary H, López-Medina C, Khan MA, Dougados M, Magrey M. Clinical profile and treatment utilisation based on HLA-B*27 status in axial spondyloarthritis: results from ASAS-PerSpA study. RMD Open. 2023;9(3):e003179. 37491128 10.1136/rmdopen-2023-003179 PMC10373694

[41] Deodhar A, Gill T, Magrey M. Human leukocyte antigen B27-negative axial spondyloarthritis: what do we know? ACR Open Rheumatol. 2023;5(7):333–44. 37222563 10.1002/acr2.11555 PMC10349221

[42] Bautista-Molano W, Navarro-Compán V, Landewé RB, Boers M, Kirkham JJ, van der Heijde D. How well are the ASAS/OMERACT core outcome sets for ankylosing spondylitis implemented in randomized clinical trials? A systematic literature review. Clin Rheumatol. 2014;33(9):1313–22. 24970597 10.1007/s10067-014-2728-6

[43] Lim CSE, Sengupta R, Gaffney K. The clinical utility of human leucocyte antigen B27 in axial spondyloarthritis. Rheumatology (Oxford). 2018;57(6):959–68. 29029331 10.1093/rheumatology/kex345

[44] Haroon M, O’Rourke M, Ramasamy P, Murphy CC, FitzGerald O. A novel evidence-based detection of undiagnosed spondyloarthritis in patients presenting with acute anterior uveitis: the DUET (dublin uveitis evaluation tool). Ann Rheum Dis. 2015;74(11):1990–95. 24928841 10.1136/annrheumdis-2014-205358

[45] Chung HY, Machado P, van der Heijde D, D’Agostino MA, Dougados M. HLA-B27 positive patients differ from HLA-B27 negative patients in clinical presentation and imaging: results from the DESIR cohort of patients with recent onset axial spondyloarthritis. Ann Rheum Dis. 2011;70(11):1930–36. 21803752 10.1136/ard.2011.152975

[46] Salinas RG, Prado ES, Ruta S. Axial involvement in psoriatic arthritis: effect on peripheral arthritis and differential features with axial spondyloarthritis in South America. J Rheumatol. 2021 Aug;48(8):1346–48. 33858975 10.3899/jrheum.201627

[47] IBGE – Instituto Brasileiro de Geografia e Estatística (Brazilian Institute of Geography and Statistics). Censo Brasileiro de 2022-2023. Rio de Janeiro: IBGE; 2023.

[48] Braun J. Why are the classification and diagnosis of axial spondyloarthritis sometimes so difficult? Rheumatology (Oxford). 2024;63(2):264–66. 37773984 10.1093/rheumatology/kead532

